# Iodine k-edge dual energy imaging reveals the influence of particle size distribution on solute transport in drying porous media

**DOI:** 10.1038/s41598-018-29115-0

**Published:** 2018-07-16

**Authors:** Salomé M. S. Shokri-Kuehni, Mina Bergstad, Muhammad Sahimi, Colin Webb, Nima Shokri

**Affiliations:** 1 School of Chemical Engineering and Analytical Science, The University of Manchester, Manchester, UK; 2 Mork Family Department of Chemical Engineering and Materials Science, University of Southern California, Los Angeles, California, 90089-1211 USA

## Abstract

Increasing salinity in groundwater and soil poses a threat to water and land resources. With the expectation of major changes to the hydrological cycle through climate change, the need for understanding the fundamental processes governing solute transport through soil has grown significantly. We provide experimentally verified insights into the influence of particle size distribution on solute transport in porous media during evaporation at the pore- and macro-scales. To do so, we utilized four-dimensional (space plus time) synchrotron X-ray tomography for iodine k-edge dual energy imaging to obtain solute concentration profiles in every single pore during saline water evaporation from coarse- and fine-grained sands. Close to the surface of the coarse-grained sand significantly higher salt concentrations were observed when compared to fine-grained sand with the same porosity under similar cumulative evaporative mass losses. The physics behind this behaviour was delineated using the recorded data with high spatial and temporal resolutions. Moreover, the measured data enabled us to quantify the variations of the effective dispersion coefficient during evaporation and how it is influenced by the particle size distribution. We show that, contrary to common assumption in modelling of solute transport during evaporation, the effective dispersion coefficient varies as a function of liquid saturation and the length of the invaded zone during evaporation from porous media, and that it increases as liquid saturation decreases.

## Introduction

Water and land quality have long been issues of global concerns. Increasing salinity in groundwater and soil poses a threat to the ecosystem functioning, water quality and evaporation, and crop production^1–6^. Understanding solute transport in the flow of water through soil entails studying the phenomenon through partially saturated zone during evaporation, which is a complex process.

During evaporation of saline water from porous media, solutes are transported to the vaporization plane via capillary-induced liquid flow, while diffusion tends to homogenise the concentration laterally throughout the pore space. The interaction between the two determines the dynamics of solute distribution in porous media^7,8^. Numerous studies have been conducted in the past to look into the effects of various parameters, such as particle size, wettability, heterogeneity, and the type of salt^9–16^, on saline water evaporation from porous media. Much less attention has, however, been paid to experimental study of how solutes are transported and distributed throughout the pore space during evaporation, and how transport properties, such as the effective dispersion coefficient D^*^ in porous media, vary as drying proceeds. As is well known, dispersion is convective mixing of two miscible fluids, which is modified by molecular diffusion, particularly in the slow zones of the pore space. The effective dispersion coefficient, which is usually used in the description of solute transport in porous media by the convective-dispersion equation (CDE), represents the combined effect of the two at the macroscale. Surprisingly, the majority of previous studies that modelled saline water evaporation from porous media either assumed a constant D^*^ or one that decreases with decreasing saturation^8,17–21^.

The focus of the present work is on providing new deep insights into the influence of particle size on the physics of solute transport in porous media during evaporation. In particular, the specific objective of this paper is to understand how the effective dispersion coefficient varies during evaporation from porous media and how it is influenced by the particle size distribution. To do so, we utilize four-dimensional (4D, space plus time) synchrotron X-ray tomography and iodine k-edge dual energy imaging in order to visualize the dynamics of solute transport in a complex pore space and quantify the variations of the effective dispersion coefficient with time and saturation. This provides us with a unique opportunity to underpin the physical mechanisms controlling solute transport and deposition in porous media during evaporation.

## Results and Discussion

### Quantitative characterisation of solute transport in drying porous media

**Figure 1:**
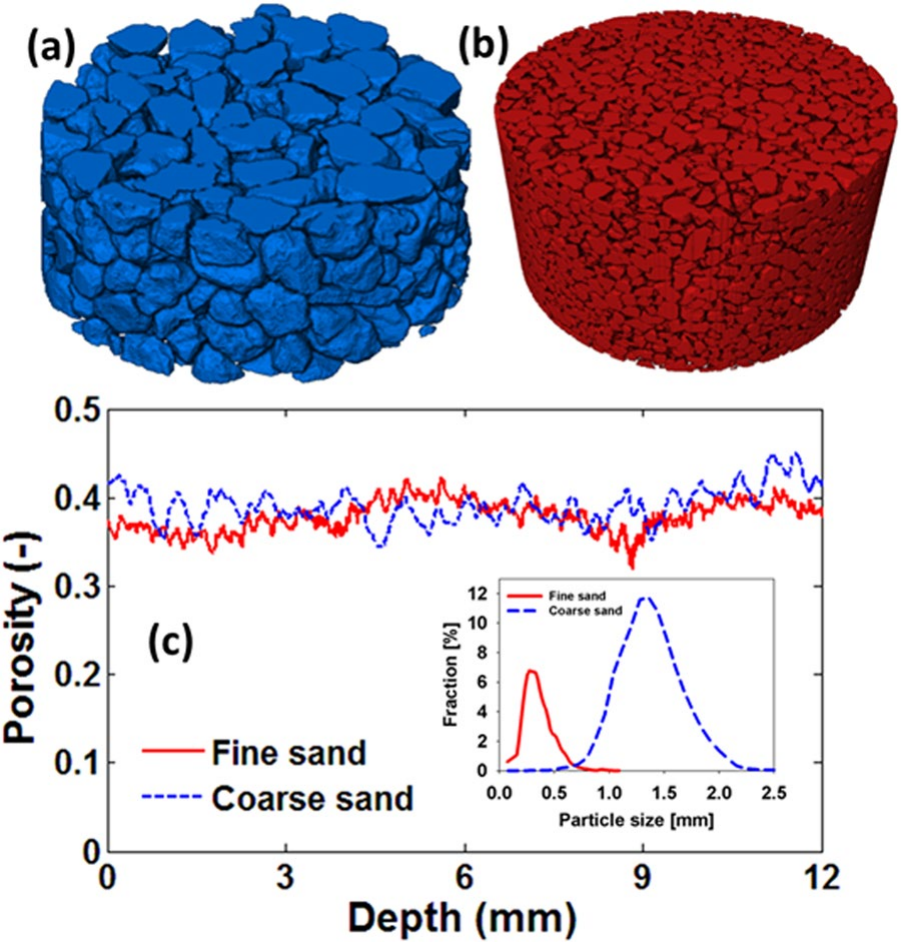
Three-dimensional rendering of the reconstructed volume of the packed (**a**) coarse and (**b**) fine sands with the corresponding porosity presented in (**c**). The inset shows the particle-size distributions of the sand grains used in the experiments.

The experimental setup and imaging details are the same as those reported by Shokri^21^. Briefly speaking, we conducted 4D synchrotron X-ray tomography experiments with fine and coarse sands, with the particle-size distributions presented in Fig. 1, packed in cylindrical columns of 8 mm diameter and 16 mm height. The columns were open to air from top for evaporation. The sand column was initially saturated with a salt solution containing 5% (by weight) calcium iodide. The dynamics of the evaporation process from the sand packs were visualized using synchrotron X-ray tomography in order to resolve the details of phase distribution and solute transport at pore-scale.

Image analysis and reconstruction were done using Avizo Fire 9.2 (FEI, 2017) and in-house codes developed in MATLAB. Further details of the experiments and image analysis are given in the Methods section below. Figure 1 shows a 3D rendering of the reconstructed volume of the packed coarse- and fine-grained sand, together with their porosity variations that were quantified using the segmented images.

**Figure 2:**
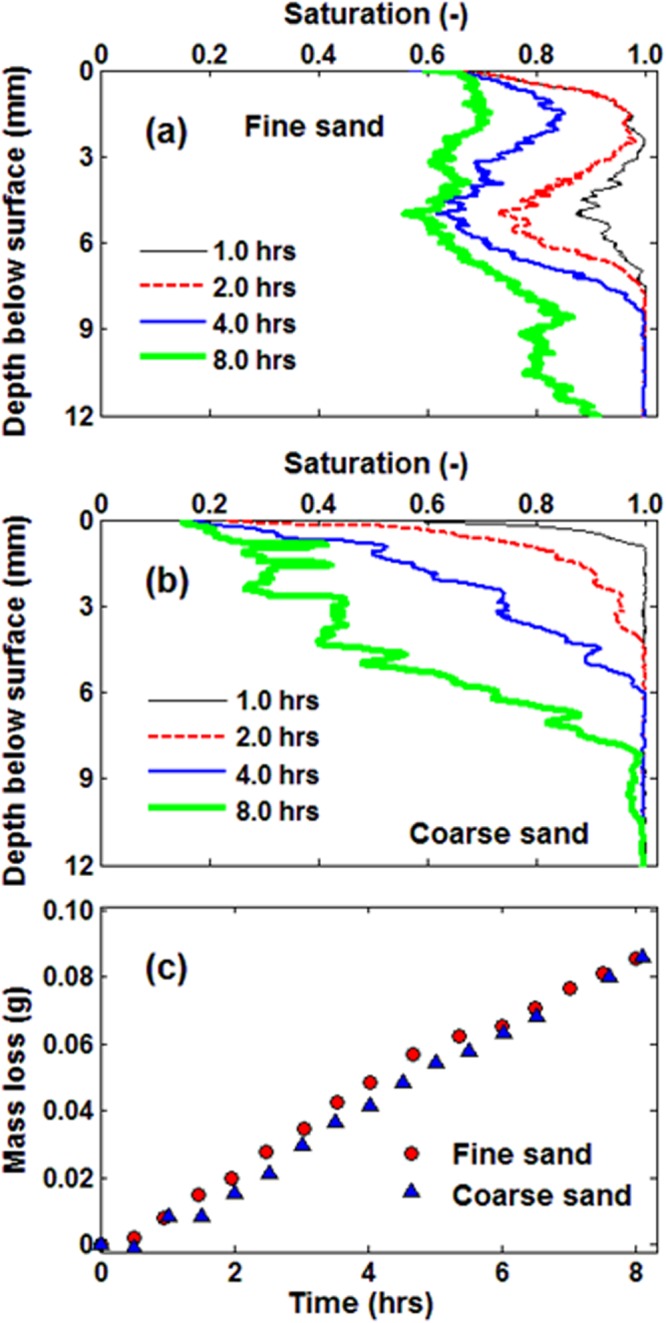
Time-dependence of the liquid saturation profiles, from the onset of the experiment (indicated in the legend) during evaporation from (**a**) fine- and (**b**) coarse-grained sands. (**c**) The computed cumulative mass losses over time during evaporation, computed using the segmented images obtained by synchrotron X-ray tomography.

The segmented images were used to calculate the water saturation in each cross section, from which the cumulative evaporative mass losses were calculated. Figure 2 shows the water saturation profiles together with the cumulative evaporative mass losses, measured during the evaporation experiments with the fine and coarse sands. The results indicate that the evaporation rates in both cases were nearly the same over the course of the experiments, despite notable differences in the measured water saturation profiles. This is expected because during early stages of the process the evaporation rate is mainly dependent on the external conditions, which were similar for both experiments. Note that the ambient temperature and relative humidity remained the same during the experiments.

The water saturation profiles depend strongly on the dynamics of the fluid front displacement during the evaporation process. The geometry and dynamics of the displacement front have been analysed in several studies^22,23^ that revealed the dependence of the front dynamics on the interplay between gravity, capillarity and viscous forces. Different pore-size distributions of the fine and coarse sands influence the capillarity forces and, consequently, the liquid-phase distribution. During water evaporation, the displaced air-water interface is pinned in regions with small pores, while continuing to recede in the regions with large pores. This results in non-uniform liquid-phase distribution over time and space. For example, Fig. 2(a) indicates that a part of the air-water interface is pinned in a region nearly 3 mm below the surface, whereas air continues to invade the medium preferentially through larger pores up to a depth of about 8 mm below the surface. Moreover, Fig. 2 confirms the presence of more water at the surface of fine-textured sand compared with coarse-textured sand. This is due to the higher air entry pressure of fine compared to coarse sand, due to the presence of smaller pores. Although the cumulative mass losses are nearly the same in the case of fine- and coarse-grained sand, the liquid phase distribution above the drying front (the interface between saturated and unsaturated zone) is remarkably different. In other words, with same evaporative mass losses, the length of the unsaturated zone (the distance over which solute is transported) is different in the fine- and coarse-grained sand. This significantly influences the dynamics of solute transport that will be discussed next.

### Solute concentration profiles

**Figure 3:**
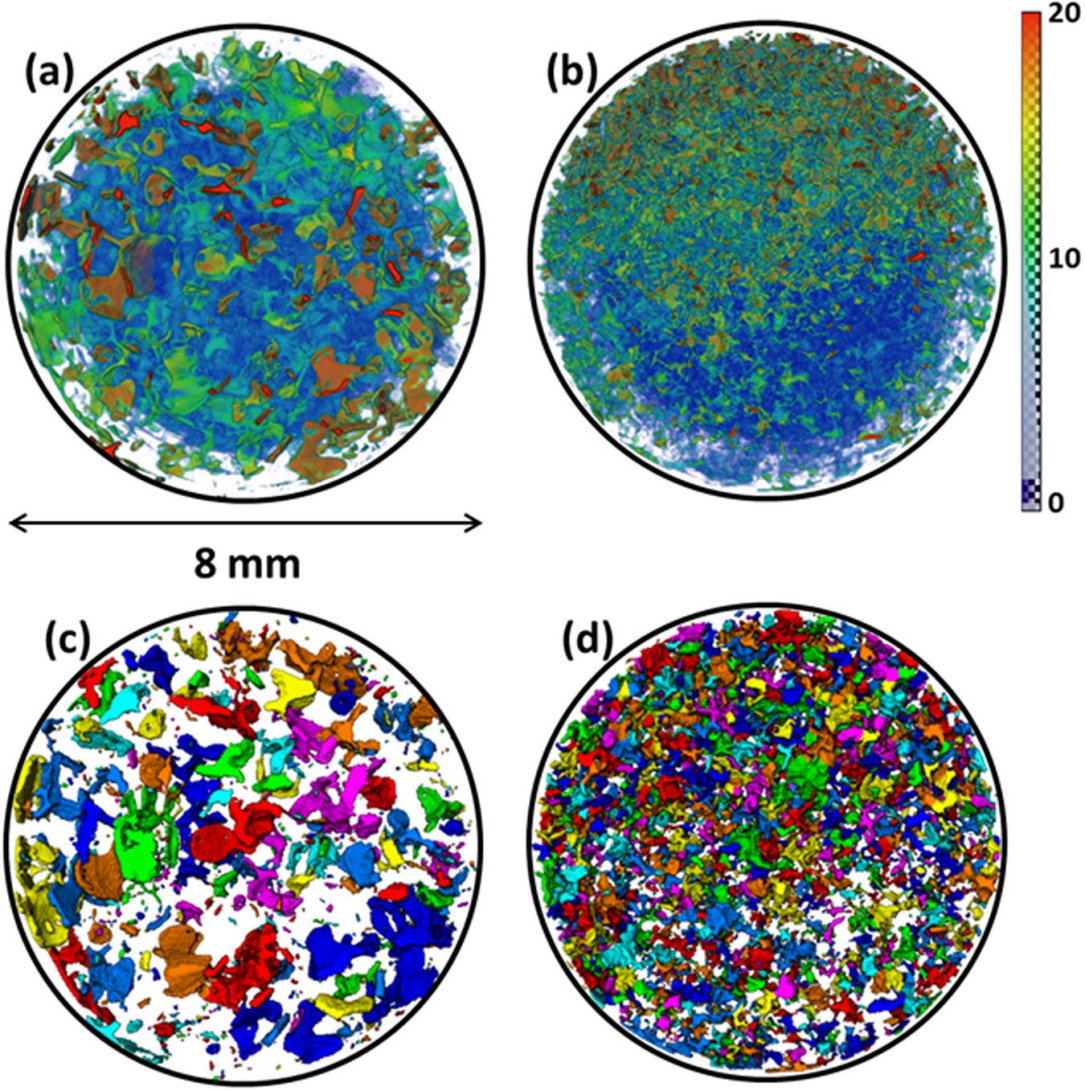
Distribution of solute concentration within the top 1 mm of (**a**) coarse- and (**b**) fine-textured sands 6 hours after the onset of the experiments. The colour map represents the solute concentration percentage, such that the closer to red, the higher the concentration. Also presented in (**c**) and (**d**) are the liquid cluster distributions within the top 1 mm of the fine- and coarse-grained sands. The clusters are defined as the liquid within pore bodies separated through pore throats. The colour represents the size of clusters.

Using iodine k-edge dual energy subtraction^21^, we obtained the salt concentration profiles with high spatial and temporal resolutions, and analysed the effect of the pore-size distribution on the saline water evaporation and variation of the effective dispersion coefficient, as defined earlier. Details of the procedure used to compute the solute concentration throughout the evaporating columns using the recorded images are presented in the Method summary section. Figure 3 shows the solute concentration distribution at the surface of the two types of sand packs 6 hours after the onset of evaporation (colours closer to red indicate higher concentrations).

Closer inspection of Fig. 3(a,b) reveals that the average solute concentration is higher at the surface of coarse-grained sand than it is with the fine-grained sand. Note that in both cases the initial concentration, porosity and, more importantly, the evaporation rate were nearly the same, yet the solute distribution at the surface of the two sand packs are significantly different. We attribute this difference to the dominant impact of the preferential liquid evaporation from finer pores at the surface, leading to non-uniform ion distribution.

In addition, the qualitative results presented in Fig. 3 indicate that at the surface of both sand packs, liquid clusters of the same sizes exhibit distinct solute concentrations. This suggests that the solute concentration depends not only on the pore/cluster sizes, but also on how the liquid clusters and patches are distributed at the surface. This happens due to the influence of the spacing between the liquid clusters on the corresponding evaporation rate per cluster^24,25^. Shahraeeni *et al*.^24^ showed that, due to the impact of the spacing between the clusters on the formation of a diffusive vapour shell above the surface, larger spacing between wet patches at the surface of porous media results in higher evaporation per pore. As described by Bergstad and Shokri^26^, such a phenomenon leads to higher salt concentrations in the wet patches, when distributed distantly. We also analysed the liquid cluster distribution within the top 1 mm of the sand columns, and the results are presented in Fig. 3(c,d). These confirm the existence of larger spacing between the liquid clusters in the coarse sand, compared to the fine packing.

**Figure 4:**
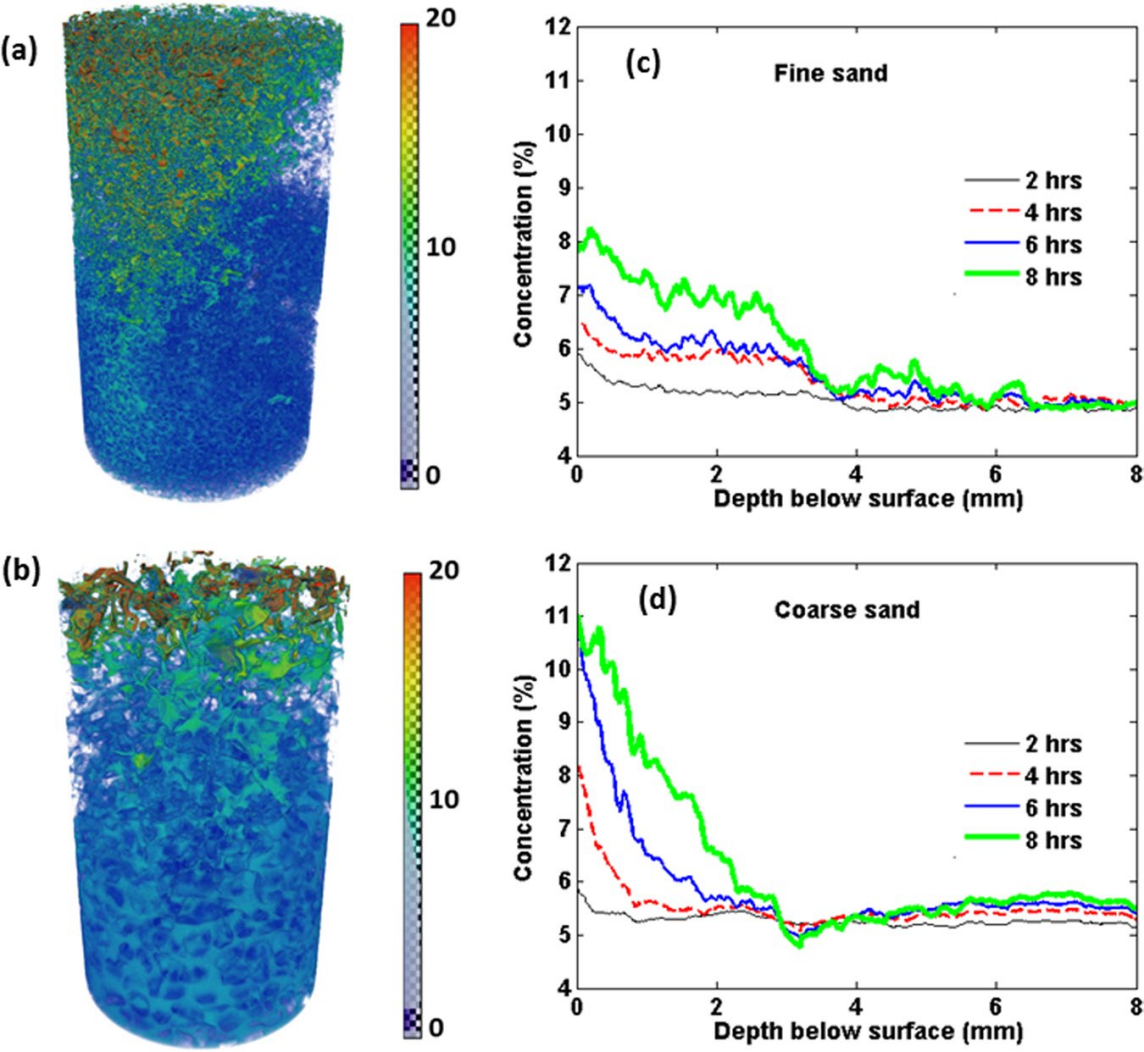
Reconstruction of the liquid phase in the top 5 mm of the (**a**) fine and (**b**) coarse sand 8 hours after the onset of evaporation with the colour map representing the solute concentration percentages. Colours closer to red indicate higher concentrations. Also presented is the dynamics of solute distribution in (**c**) fine and (**d**) coarse sand at various times from the onset of the experiments (indicated in the legend).

Using the 4D images recorded by synchrotron X-ray tomography, we calculated the solute concentration along the sand profiles over time. The results are presented in Fig. 4, illustrating a sharper gradient of solute concentration closer to the surface of the coarse sands compared with the fine sand. Typical examples are presented in Fig. 4(a,b) that illustrate, respectively, the solute concentration after 8 hours of evaporation in the case of fine and coarse sands.

Although both porous media have similar porosity and initial salt concentration, and were placed under similar evaporative demand, the pore-scale solute distributions through the two evaporating sand columns are remarkably different. Such pore-scale phenomena affect the macroscopic response, presented in Fig. 4(c,d). The results indicate that, relative to the coarse sand, the solute is distributed more uniformly through the fine sand packing. This suggests that the effective dispersion coefficient is higher in the fine sand. As illustrated in Fig. 4(a,b), there exist more tortuous capillary pathways in the fine sand. This leads, as a result of the existence of more junctions within the complex liquid network and the longer lengths of the flow paths, to more mixing, implying higher effective dispersion coefficients that influence the solute transport during evaporation.

### Estimating the dispersion coefficient

Under the assumptions of negligible solute adsorption on the solid surface and a 1D vertical solute transport, the equation that governs solute transport in the evaporating liquid confined to porous media is given by^7,18^:

1 $$\frac{\partial (\rho \varepsilon SC)}{\partial t}=\frac{\partial }{\partial z}(\rho \varepsilon S{D}^{\ast }\frac{\partial C}{\partial z}-\rho \varepsilon SCU)$$

where *C*(*z*, *t*), *ε*, *ρ*, *t*, *z* and *S* indicate, respectively, the solute mass fraction, porosity, density of the solution, elapsed time from the onset of the evaporation, depth below the surface, and the liquid saturation. *U* corresponds to the average liquid velocity and D^*^ is the effective dispersion coefficient of the solute in porous media, representing the combined effect of mixing by convection and diffusion. As mentioned earlier, in many previous studies in which Eq. (1) was used to describe solute transport in porous media during evaporation, D^*^ was assumed to be either constant or decreasing with decreasing liquid saturation^8,17–21^. Using the experimental pore-scale information, we investigate the variation of D^*^ during saline water evaporation from porous media.

**Figure 5:**
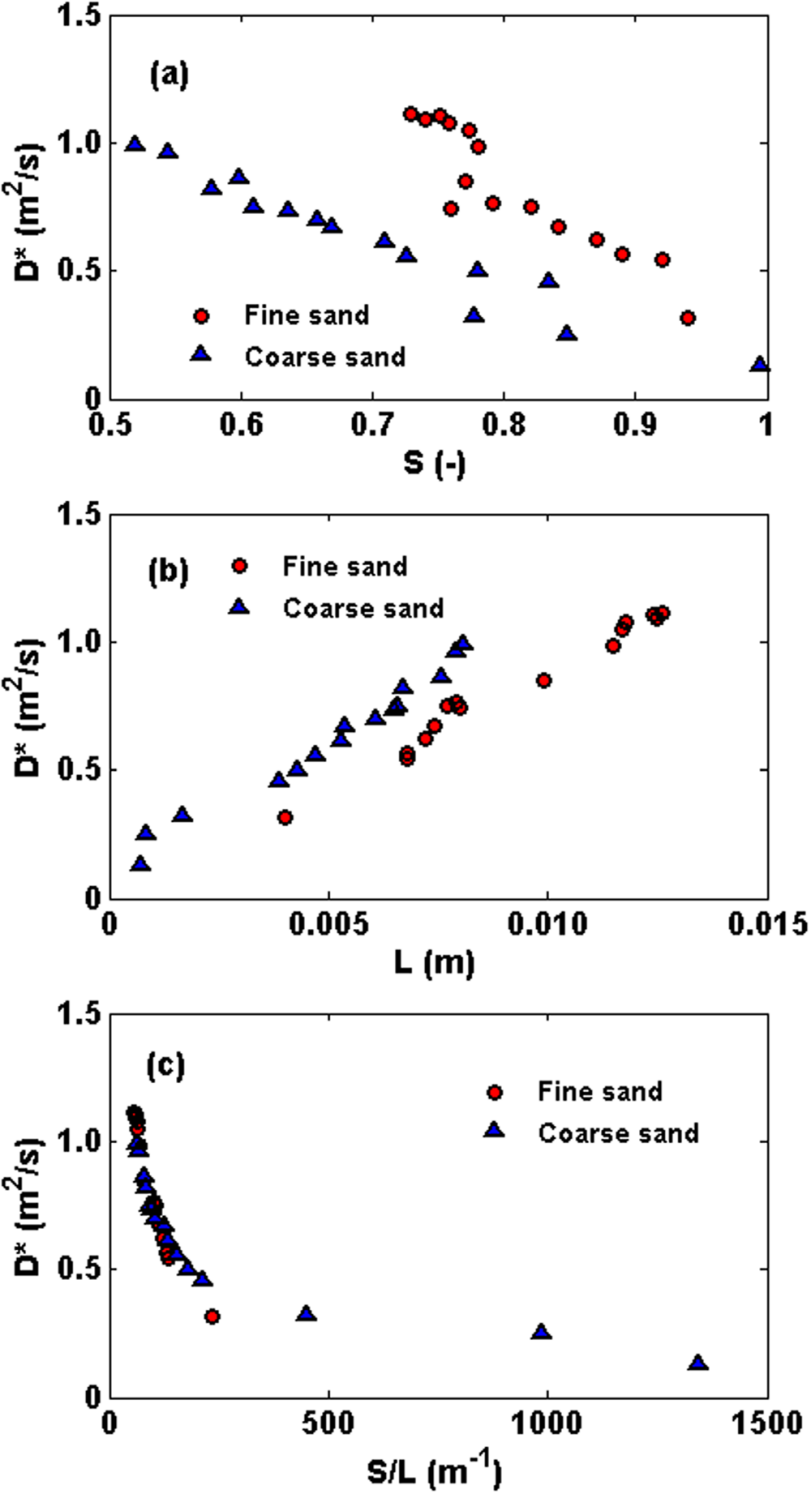
The variation of D^*^ versus (**a**) the average saturation of the invaded zone *S*, (**b**) length of the invaded (or unsaturated) zone *L* and (**c**) *S/L*.

We utilized the analytical solution developed by Guglielmini *et al*.^7^ to estimate D^*^ using the measured salt concentration profiles. Guglielmini *et al*.^7^ developed the following analytical solution to describe the dynamics of solute concentration in drying porous media at intermediate times:

2 $${\rm{\Omega }}({\xi },{\rm{\tau }})=1-{\rm{Pe}}\tau +\frac{{{\rm{Pe}}}^{2}\,\tau \,{\rm{erfi}}(\frac{\sqrt{{\rm{Pe}}}(\xi -1)}{\sqrt{2-2Pe\tau }})}{({\rm{Pe}}\tau -1){\rm{erfi}}(\frac{\sqrt{{\rm{Pe}}}}{\sqrt{2-2{\rm{Pe}}\tau }})}$$

where erfi is the imaginary error function, Ω is the dimensionless effective solute density at dimensionless depth *ξ* below the surface, with *ξ* defined as $\xi =z/L$. Here, *L* is the length scale over which the solute transport occurs, which is the length of invaded (or unsaturated) zone, *τ* is the dimensionless time defined as, $\tau =t{D}^{\ast }/{L}^{2}$. In Eq. (2), *Pe* represents the Peclet number defined as ${\rm{Pe}}=\frac{Le}{\rho \varepsilon {D}^{\ast }}$ with *e* representing the evaporation rate. The analytical solution was obtained with the assumption of constant *S* and D^*^. In our experiments, for a given saturation the system is fixed, hence the analytical solution is applicable to the state of the system at that saturation for a given scan. This allowed for a semi-quantitative estimate of the effective dispersion coefficient by fitting the analytical solution given by Eq. (2) to the measured concentration profiles. An example of fitting Eq. (2) to the experimentally-determined concentration profiles is presented in the Supplementary Information. The computed D^*^ in the fine and coarse sands are reported in Fig. 5 as a function of (a) average saturation of the invaded zone *S*; (b) the length *L* of the invaded zone, and (c) *S/L*. The results presented in Fig. 5 show that D^*^ increases during evaporation as liquid saturation decreases. This is contrary to the assumption made in previous studies regarding a constant effective dispersion coefficient or one that decreases with decreasing saturation in drying porous media^8,17–21^. The inverse relationship between D^*^ and liquid saturation indicated by Fig. 5 suggests that D^*^ in Eq. (1) should be treated as an effective dispersion coefficient when this equation is used to model saline water evaporation from porous media.

That the effective dispersion coefficient increases over time is due to the fact that, as the evaporation process proceeds, water saturation decreases. Therefore, the flow paths become increasingly more tortuous and longer, hence extending the mixing zone considerably. This leads to better mixing over longer distances, resulting in larger effective dispersion coefficients. The increase in D^*^ at lower liquid saturations was first predicted by Sahimi *et al*.^27–29^, and was subsequently confirmed in numerous experiments with various types of porous media^30–32^. Although this inverse relationship between D^*^ and the liquid saturation is relatively well-established and confirmed in one- or two-phase fluid displacement processes in porous media, to the best of our knowledge, a systematic analysis for delineating the relationship between D^*^ and liquid saturation in porous media during evaporation was not attempted before.

Figure 5 indicates that the effective dispersion coefficient changes almost one order of magnitude over the duration of the experiments, and that it does not necessarily change linearly with the liquid saturation, a main assumption made commonly in literature. In fact, as was first shown by Sahimi and Imdakm^33^, percolation theory^34,35^ predicts that as the water saturation decreases and approaches its residual value at which it becomes disconnected, D^*^ increases as a power law in (S-*S*_*r*_), where *S*_*r*_ is the residual saturation, with the exponent of the power law estimated by Sahimi and Imdakm^33^ to be about (−0.2) in 3D and (−2.8) in 2D porous media.

We found a strong linear relationship between D^*^ and the length of the invaded zone. Since the dispersivity, the ratio of the dispersion coefficient and mean flow velocity, is proportional to the length of the invaded zone, the implication is that the average macroscopic velocity remains essentially constant. As the drying front - the interface between the saturated and unsaturated zones - recedes into the porous medium, the length of the invaded zone increases. This leads to the formation of a more tortuous liquid network that contributes towards better mixing and, thus, higher D^*^. Thus, the data confirm that D^*^ depends not only on the saturation, but also on the complex liquid network and its morphology formed during the evaporation process.

Note that, due to the receding drying front during evaporation from porous media, the length over which solute travels increases with time. The liquid saturation above the drying front depends on the pore-size distribution of porous media (among other factors). Spatial heterogeneity of pore-sizes promotes preferential air invasion through larger pores and therefore, two porous media with different particle-size distribution may have similar saturation above the drying front, but very different length of the unsaturated (invaded) zone. For example, in our experiments, under a constant liquid saturation above the drying front, the length of the unsaturated zone is longer in fine-grained sand compared to the coarse-grained sand, hence resulting in longer solute travel distances which leads to more dispersion in the former case under similar average saturation above the drying front. Moreover, when the length of unsaturated zone is the same in coarse- and fine-grained sand, the saturation in coarse sand is less resulting in more tortuous and longer flow paths than in the fine sand which leads to more dispersion in the coarse sand as illustrated in Fig. 5(b). This suggests that evaluating D^*^ solely based on the average saturation may not capture the entire physics that controls solute transport in porous media during evaporation, and that one should simultaneously take into account the drying front depth, the length over which solute transport occurs. Based on the data presented in Fig. 5(a,b), we looked into the relationship between D^*^ and the combined effects of saturation of the invaded zone and the length of the invaded zone, expressed as *S/L*. The results are presented in Fig. 5(c).

Nearly similar variation of D^*^ in both fine and coarse sand, when plotted versus *S/L*, suggests that the two parameters, the average saturation of the invaded zone and the length of the invaded zone, are the key factors influencing D^*^ in drying porous media.

The present analysis extends the physical understanding of the mechanisms governing solute transport in porous media during evaporation and how the parameters such as particle size distribution or permeability of porous media influences solute transport in drying porous media. Moreover, the data and analysis are relevant to characterisation of drying of droplets containing solutes or dispersed particles with the associated deposition patterns^36,37^.

## Methods Summary

We conducted synchrotron X-ray microtomography evaporation experiments with fine and coarse sands, initially fully saturated with a salt solution containing 5% calcium iodide (by weight) and packed in cylindrical plastic columns with an inner diameter of 8 mm and height of 16 mm. The experiments were conducted on the GeoSoilEnviroCARS (GSECARS) BM-13BMD beamline at the Advanced Photon Source, Argonne National Laboratory, IL. The columns were closed at all boundaries, except at the top that was open to air for water evaporation. The external conditions - ambient temperature and relative humidity - were similar and constant in both experiments. Image reconstruction was performed using programs developed by GSECARS to convert X-ray attenuation to 3D volumetric data. The resolution of the images was 12.01 microns. In this analysis, the top 13.2 mm of the sand column is used that includes 1103 reconstructed images of the 2D horizontal cross sections, with grey-scale values representing density distribution within the porous medium. The columns were scanned every 30 minutes during drying. The sand packs were visualized twice, once with X-rays with energies immediately above the K-edge (33.1694 keV) value of Iodide, and a second time with X-rays with energies immediately below the K-edge value at 33.0690 keV. The difference in the grey value of the two scans yields the salt concentration at pore scale.

To convert the gray value to salt concentration, we followed the calibration method described by Shokri^21^. Briefly, to calibrate the gray values, CaI_2_ solutions with concentration of 2%, 5%, and 25% (by mass) were imaged at the same energy levels above and below the Iodine K-edge value. A linear equation was obtained relating the known values of concentrations to the corresponding gray values of the solutions with a well-defined concentration. The linear equation was used as the calibration curve to relate the gray value at any given pixel and time during evaporation from sand columns to the salt concentration. This enabled us to delineate the temporal and spatial solute distribution at the pore-scale during evaporation.

Moreover, to quantify the liquid phase distribution and how it is influenced by the particle size distribution, the recorded pore-scale images were segmented following the procedure described by Shokri^21^ and Shokri *et al*.^38^. In-house codes were developed in MATLAB to analyse the images and distinguish between liquid, air and solid phases in each 2D cross section according to the distribution of their gray values. Threshold values were calculated to segment each phase. The segmented images were used to quantify the drying front displacement in porous media, the 3D dynamics of liquid phase distribution and the evaporative mass losses (presented in Fig. 2(c)). We used several segmentation algorithms for image analysis and liquid phase quantification and the estimated errors were at most as large as the size of the symbols in Fig. 2(c).

### Data availability

The data presented in this manuscript will be available freely via sending a request to the corresponding author.

## Electronic supplementary material

SUPPLEMENTARY INFO
