# Supplementary material for: Iodine k-edge dual energy imaging reveals the influence of particle size distribution on solute transport in drying porous media
Source: Sci Rep. 2018 Jul 16;8:10731. doi: 10.1038/s41598-018-29115-0 (PMC6048136; doi:10.1038/s41598-018-29115-0)
Supplement: Supplementary file 1 — SUPPLEMENTARY INFO [file 41598_2018_29115_MOESM1_ESM.docx]

**Iodine k-edge dual energy imaging reveals the influence of particle size distribution on solute transport in drying porous media**

Salomé M.S. Shokri-Kuehni (1), Mina Bergstad (1), Muhammad Sahimi (2), Colin Webb (1), Nima Shokri (1)*

(1) School of Chemical Engineering and Analytical Science, The University of Manchester, Manchester, UK

(2) Mork Family Department of Chemical Engineering and Materials Science, University of Southern California,Los Angeles, California 90089-1211, USA

**Supplementary information**

Fig. S1 illustrates a typical example of the fitting the analytical model, Eq. (2), to the experimentally determined concentration profile to estimate the effective diffusion-dispersion coefficient presented in Fig. 5.


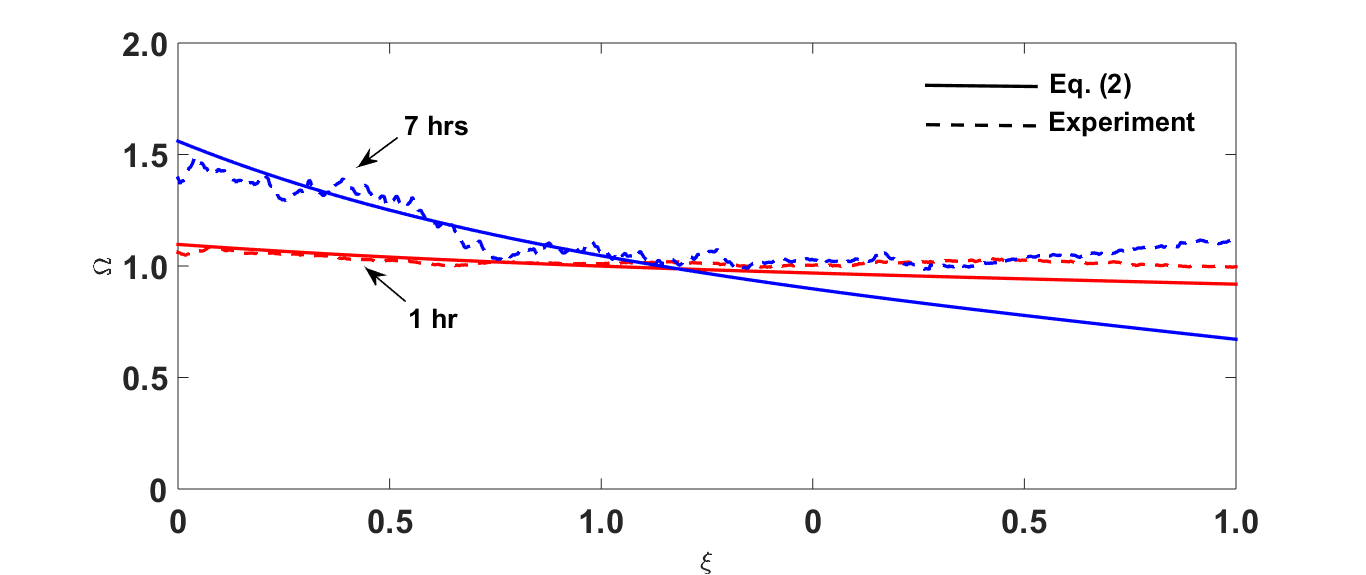


**Figure S1.** An example of fitting Eq. (2) (solid lines) to the concentration profiles computed using the recorded pore-scale images (dash lines). The concentration profiles correspond to the fine-grains sand after 1 and 7 hrs from the onset of the evaporation experiments.
